# The *Caenorhabditis elegans* GATA Factor ELT-1 Works through the Cell Proliferation Regulator BRO-1 and the Fusogen EFF-1 to Maintain the Seam Stem-Like Fate

**DOI:** 10.1371/journal.pgen.1002200

**Published:** 2011-08-04

**Authors:** Charles Brabin, Peter J. Appleford, Alison Woollard

**Affiliations:** Department of Biochemistry, University of Oxford, Oxford, United Kingdom; University of Wisconsin, United States of America

## Abstract

Seam cells in *Caenorhabditis elegans* provide a paradigm for the stem cell mode of division, with the ability to both self-renew and produce daughters that differentiate. The transcription factor RNT-1 and its DNA binding partner BRO-1 (homologues of the mammalian cancer-associated stem cell regulators RUNX and CBFβ, respectively) are known rate-limiting regulators of seam cell proliferation. Here, we show, using a combination of comparative genomics and DNA binding assays, that *bro-1* expression is directly regulated by the GATA factor ELT-1. *elt-1(RNAi)* animals display similar seam cell lineage defects to *bro-1* mutants, but have an additional phenotype in which seam cells lose their stem cell-like properties and differentiate inappropriately by fusing with the hyp7 epidermal syncytium. This phenotype is dependent on the fusogen EFF-1, which we show is repressed by ELT-1 in seam cells. Overall, our data suggest that ELT-1 has dual roles in the stem-like seam cells, acting both to promote proliferation and prevent differentiation.

## Author Summary

Stem cells can both produce differentiated cells and self-renew, producing more stem cells. Choosing between these opposing options is critical for development. Here, we have investigated the molecular genetics underlying this choice in the nematode worm, *C. elegans*, using the seam cells as a model of stem cell divisions. The transcription factor RNT-1 works together with BRO-1 (homologues of mammalian RUNX and CBFβ genes, respectively) to regulate proliferation of the seam cells, reflecting the roles of RUNX/CBFβ in mammalian stem cells. To better understand how *bro-1* is regulated, we looked for conserved regions of non-coding DNA, likely to be of functional importance. We identified a 122 bp conserved non-coding element that is necessary and sufficient for *bro-1* expression. Subsequent analysis suggested that the GATA transcription factor ELT-1 directly regulates *bro-1*. We have found that ELT-1 actually performs two distinct roles, promoting proliferation of seam cells while also preventing them from inappropriately fusing with surrounding tissue and losing their stem-like properties. Furthermore, we propose a link between the retention of stem cell properties and the maintenance of seam cells in a distinct compartment, in which they are protected from differentiation.

## Introduction

The regulation of the decision between cell proliferation and differentiation is a key aspect of metazoan development, ensuring that the correct number and type of cells are present. The RUNX family of transcription factors (*RUNX1*, *2* and *3*), together with their binding partner CBFβ, are key players in the control of stem cell proliferation in haematopoiesis [1]–[3], osteogenesis [4]–[6] and neurogenesis [7], [8]. Moreover, mutations in these genes are known to cause a variety of diseases, with both CBFβ and RUNX genes having the potential to act as either oncogenes or tumour suppressors, depending on the nature of the mutations and the context in which they act [9].

In the nematode worm, *Caenorhabditis elegans*, RUNX and CBFβ are each represented by a single gene, *rnt-1* and *bro-1* respectively. Reflecting their role in mammalian tissues, RNT-1 and BRO-1 are involved in the regulation of the division patterns of the stem cell-like seam cells [10]–[12]. The seam comprises a specialized epithelial tissue of lateral hypodermal cells located along each side of the worm and provides a tractable model system for studying the balance between proliferative and differentiative developmental decisions. The seam cells are considered stem cell-like because of their ability to both self-renew and to produce a variety of differentiated cell types; in addition to increasing the number of seam cells during some divisions, the lineage contributes cells to the hypodermis (an epithelial tissue), as well as giving rise to neurons and glial cells [13]. Seam cells do not self-renew throughout adult life, and have not yet been shown to reside in a classic “niche”, hence the qualification stem-like, but they do provide a useful simplified model for the stem cell mode of division throughout larval development that is well established [14]–[21].

The choice between the two very different developmental alternatives of proliferation and differentiation is intrinsically linked to the way in which the seam cells divide. Symmetrical divisions allow the expansion of the stem cell pool, as both daughters of the division retain the seam (stem cell-like) fate. Conversely, asymmetrical divisions initiate developmental pathways that result in the production of differentiated cell types. Whilst one daughter of the division (the posterior daughter, in the case of cells V1-6 in the seam lineage) retains the seam fate, the other loses its ability to proliferate further and instead proceeds to differentiate into one of a number of different cell types, most commonly assuming the hypodermal fate and fusing with the hyp7 syncytium. In this case, the differentiation event involves fusion with a different cell type, although seam cells can differentiate independently of fusion, for example taking on neuronal fates [13]. At the end of larval development, seam cells undergo homotypic fusion, producing the fully differentiated adult seam cell syncytium that secretes the alae [13]. The seam cells divide repeatedly throughout larval development, and the form that the division takes (symmetrical or asymmetrical) is critically dependent on the developmental stage of the worm. The nature of these stem cell-like divisions, and thus the fate of the daughter cells arising from them, therefore requires precise regulation both spatially and temporally.

In *C. elegans*, *rnt-1* and *bro-1* are rate-limiting regulators of seam cell divisions, with both genes being required for proliferation in this tissue. In *rnt-1* and *bro-1* mutants, the number of seam cells present in individuals is reduced due to division failures within the seam lineage [12]. Furthermore, over-expression of *rnt-1* and *bro-1* results in hyperplasia of the seam [10]. In this study, we have examined the mechanisms underlying the regulation of *bro-1* expression. We identified a small (122 bp) conserved non-coding element (CNE) within the first intron of *bro-1*, which we show to be both necessary and sufficient for *bro-1* expression in the seam. In order to identify direct upstream regulators of *bro-1*, a yeast one-hybrid screen was performed using this conserved element as bait. This, coupled with an *in vitro* binding assay, demonstrates that the GATA transcription factor ELT-1 directly regulates *bro-1* through this CNE. The involvement of both GATA and RUNX/CBFβ factors in regulating a stem cell-like lineage in *C. elegans* thus mirrors the situation in mammalian systems, where the activities of these proteins are intricately linked with the specification of stem cell populations (for example haematopoietic stem cells [22]).

We then sought to further investigate the role of ELT-1 in seam cells. Analysis of the *elt-1* RNAi phenotype suggests that ELT-1 has both *bro-1*-dependent and independent functions in the seam. ELT-1 functions through *bro-1* to promote proliferation of seam cells and through *eff-1* to repress inappropriate differentiation and fusion with the hypodermal syncytium. We also show that apical junction components themselves are important for the maintenance of seam cell fate, providing the correct contacts and therefore creating an environment in which cells are protected from differentiation signals emanating from surrounding tissues. Any disruption to the continuity of this environment contributes to the initiation of differentiation and loss of the seam fate.

## Results

### The first intron of *bro-1* contains a highly conserved sequence element that is both necessary and sufficient for *bro-1* expression in seam cells

**Figure 1:**
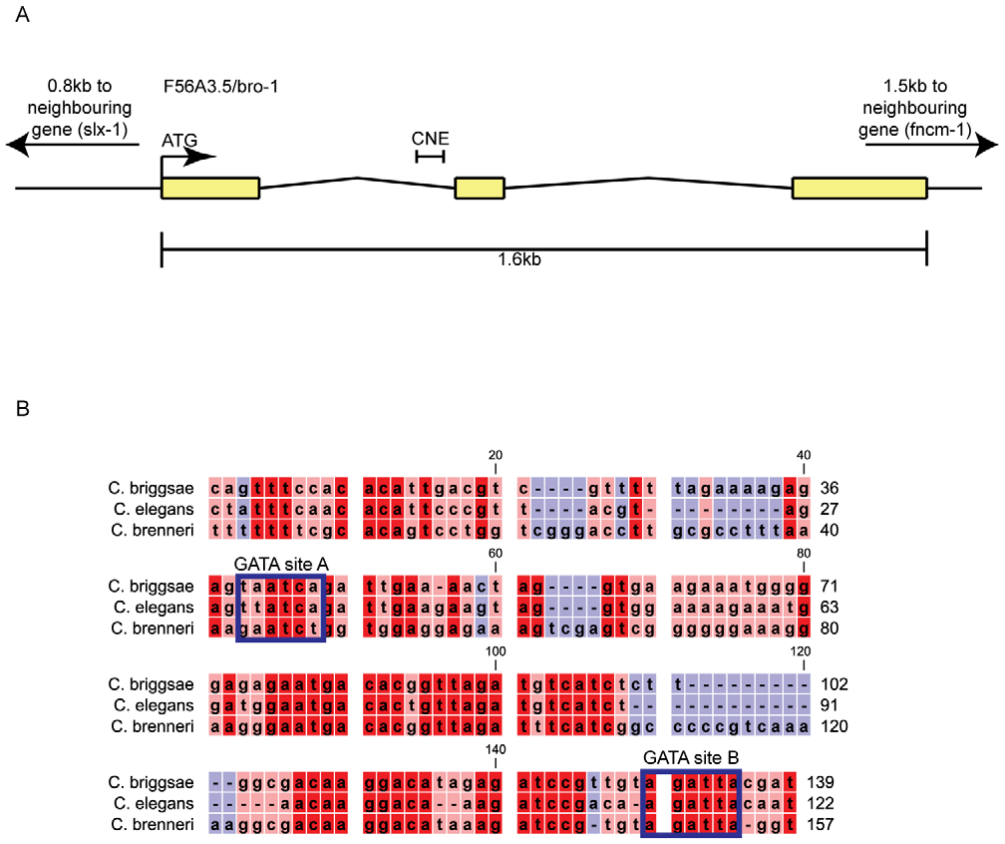
*bro-1* contains a conserved non-coding element within the first intron. (A) Genomic structure of *bro-1*, showing the size and position of the CNE. (B) Alignment of the *C. elegans bro-1* CNE with orthologues from *C. briggsae* and *C. brenneri* reveals high levels of sequence conservation at the 3′ end of intron 1. Conservation is still evident, though less pronounced, when *C. remanei* and *C. japonica* are included in the alignment (data not shown). Predicted GATA binding sites are labelled. Sequence alignments were performed using CLC Sequence Viewer v6.4 (CLC bio, Aarhus, Denmark). Red shading denotes that sequences are conserved across all 3 species of nematode; pink denotes conservation in 2 out of 3 species; blue denotes no conservation.

Comparative sequence analysis revealed high levels of conservation between exonic regions of *bro-1* in *C. elegans* and *C. briggsae* (73, 85 and 75% identity for exons 1, 2 and 3 respectively, compared to an average of 53% identity for introns 1 and 2). In contrast, we found no significant blocks of conservation in the 0.8 kb between *bro-1* and the next upstream gene. However, a 122 bp region within the first intron was found to be highly conserved (3 species alignment shown in Figure 1B) (69% identity compared to 48% for the rest of the introns). We termed this the “*bro-1* Conserved Non-coding Element” (*bro-1* CNE).

**Figure 2:**
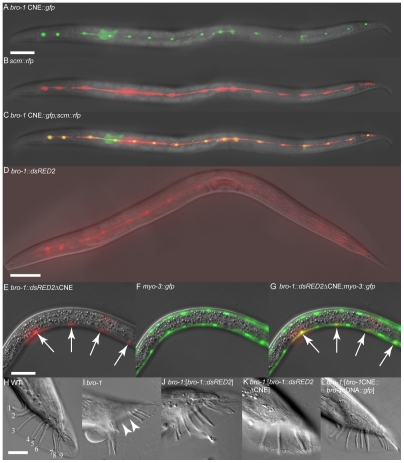
The *bro-1* CNE is both necessary and sufficient for correct expression of *bro-1*. (A–C) Transgenic animal expressing *scm::rfp* [10] and *bro-1* CNE::*gfp*. The co-localisation of these two reporters in seam cells demonstrates that the 122 bp CNE is sufficient to drive seam cell expression of GFP when present as a single copy upstream of the *pes-10* minimal promoter in plasmid *pPD107.94* (containing an NLS). RFP, driven by *scm::rfp*, localises to both the nucleus and cytoplasm of seam cells. A = *bro-1* CNE::*gfp* only, B = scm::*rfp* only, C = merge. (D) *bro-1::dsRED2* expression in the seam cells of a transgenic hermaphrodite. (E–G) Transgenic animal expressing the *bro-1::dsRED2* construct with the CNE deleted and *myo-3::gfp* to mark body wall muscle cells. A = *bro-1::dsRED2*ΔCNE only, B = *myo-3::gfp* only, C = merge. Deletion of the CNE abolishes seam expression of *bro-1* and results in high levels of expression in body wall muscle (arrows), where *bro-1* is usually expressed only very weakly. dsRED2 expression is variably absent from some nuclei owing to mosaicism of the extrachromosomal array; the *myo-3::gfp* marker is integrated. Scale bars for A–G, 50 µm. (H) WT male tail with 18 sensory rays, 9 on each side (numbered 1–9). (I) *bro-1* male tail, exhibiting missing rays (arrowheads). (J) *bro-1* male tail, rescued by full length *bro-1::dsRED2*. (K) Transgenic *bro-1* animal expressing *bro-1::dsRED2*, from which the CNE has been deleted. No rescue of the male tail phenotype is observed. (L) *bro-1* male tail, rescued by *bro-1 cDNA::gfp* driven by the CNE. Scale bars for H–L, 20 µm.

Next, we tested the ability of the *bro-1* CNE to act as an enhancer for cell-specific expression. When used in conjunction with the *pes-10* minimal promoter (*bro-1* CNE::*gfp*), the CNE was able to drive seam cell-specific GFP expression in worms (Figure 2A–2C). A vector containing a similar-sized fragment of a different, non-conserved region of intron 2 failed to drive any discernable GFP expression (data not shown). Furthermore, when the intergenic region between *bro-1* and the nearest upstream gene was used, no GFP expression was evident (data not shown). To further characterise the importance of the CNE in regulating *bro-1* expression, we first deleted the 122 bp region from the *bro-1::dsRED2* construct [10]. The wild type construct (containing the full *bro-1* genomic region) drives expression in seam cells and rescues the *bro-1* mutant male tail phenotype caused by division failures in V and T lineage seam cells (Figure 2J). In contrast, the mutagenised *bro-1::dsRED2* construct did not express in seam cells, and was unable to rescue the *bro-1* male tail phenotype (Figure 2K). Secondly, we used the CNE (together with the *pes-10* minimal promoter) to drive expression of *bro-1 cDNA::gfp* (see Text S1 and [23]). This construct drove seam cell expression, and rescued the *bro-1* mutant phenotype (Figure 2L). Thus, the *bro-1* CNE is both necessary and sufficient for *bro-1* expression in seam cells.

### The GATA transcription factor ELT-1 interacts directly with the *bro-1* CNE

Next, we used a yeast one-hybrid system to determine which transcription factors are able to bind to the *bro-1* CNE. Three tandem copies of the CNE were used as bait and screened with a mixed stage *C. elegans* transcription factor cDNA library. Of 120 positive colonies, 110 contained the clone encoding the GATA factor ELT-1, demonstrating that ELT-1 protein binds to the *bro-1* CNE in this assay.

**Figure 3:**
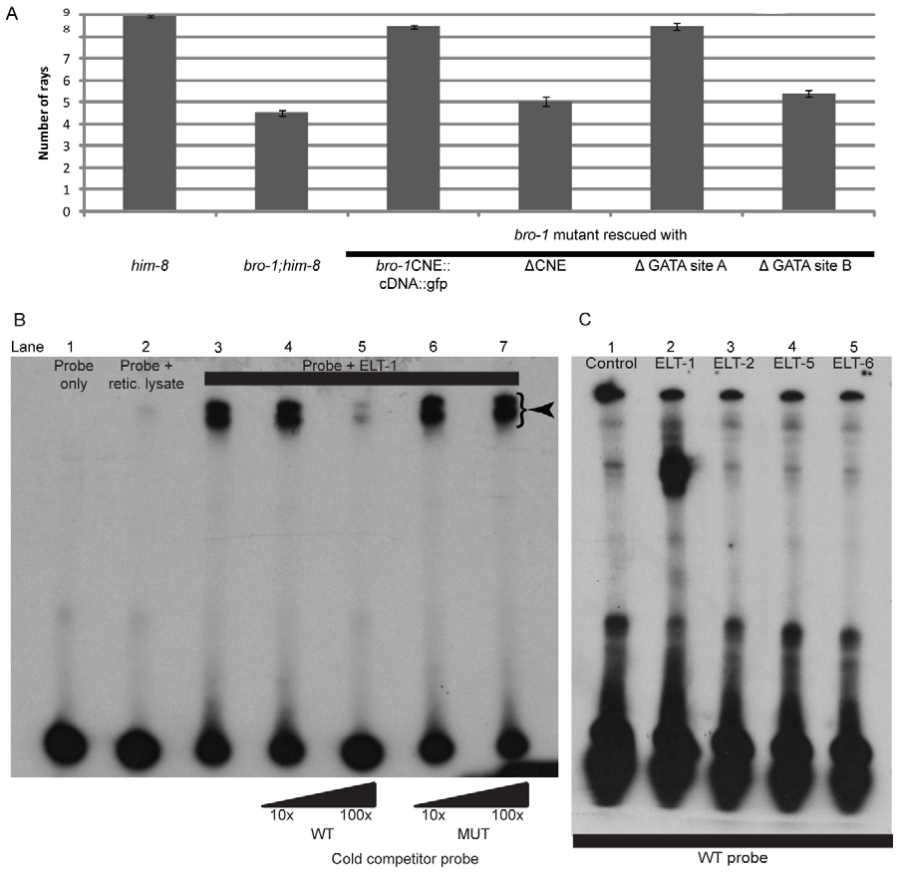
Correct *bro-1* expression depends on ELT-1 binding to GATA site B. (A) Bar chart showing ray number in the following strains: *him-8*, *bro-1;him-8*, *bro-1*;*him-8*; *bro-1CNE*::*bro-1cDNA*::*gfp*, *bro-1*;*him-8*;*bro-1ΔCNE*::*dsRED2*, *bro-1*;*him-8*;*bro-1CNEΔGATA site A*::*bro-1cDNA*::*gfp*, *bro-1*;*him-8*;*bro-1CNEΔGATA site B*::*bro-1cDNA*::*gfp*. Deletion of GATA site A has no effect on the ability of the *CNE::bro-1 cDNA* construct to rescue the *bro-1* mutant tail phenotype, while deletion of site B abolishes the rescuing ability of the construct. Error bars show SEM. (B) EMSA showing that *in vitro* translated ELT-1 shifts labelled probe (lane 3, DNA-protein complexes labelled with an arrowhead), which is not shifted on its own or in the presence of reticulocyte lysate only (lanes one and two respectively). Non-labelled WT cold competitor probe at 100× concentration (lane 5) significantly reduces the intensity of the shifted bands. Addition of mutated competitor probe has no such effect at 100× concentration (lane 7), demonstrating that the ELT-1-CNE interaction is dependent on GATA site B, which is altered in the mutated probe. (C) The interaction between ELT-1 and the *bro-1* CNE is specific. Incubation of ELT-1 protein with labelled probe results in a shift (lane 2) relative to the control incubation (lane 1). Incubation of other GATA transcription factors (ELT-2, ELT-5 and ELT-6) with the labelled probe resulted in no shift. ELT-3 was not tested as *in vitro* transcription and translation proved problematic but, like ELT-2, ELT-3 is not expressed in the seam [61] so would not bind to *bro-1* in seam cells, and was not detected amongst positive clones in the yeast one-hybrid screen, despite being present in the TF library used.

The bioinformatics software Patch [24] revealed the presence of possible binding sites for GATA transcription factors in the CNE (Figure 1B), two of which are relatively well conserved across *Caenorhabditis* species [25], [26]. Site A matches the GATA consensus sequence WGATAR, while site B is slightly different (AGATTA), but matches the target sequence for the human GATA family member GATA6 [26]. To investigate the significance of these sites, both were deleted separately from the *bro-1::dsRED2* rescuing construct. While deletion of site A had no effect on tail rescuing ability, deletion of site B abolished the ability of the construct to rescue *bro-1* mutant tails (Figure 3A).

### ELT-1 specifically binds GATA site B within the *bro-1* CNE

To confirm this interaction, an Electrophoretic Mobility Shift Assay (EMSA) was performed using *in vitro* translated ELT-1 protein and a portion of the *bro-1* CNE containing GATA site B as a probe. ELT-1 protein was able to bind the CNE, resulting in a shift in the position of the labelled probe (Figure 3B). Cold competitor probe was able to compete with the identical labelled probe, resulting in a diminution in intensity of the shifted band. However, cold probe with a mutation in GATA site B (AGATTA to ATAGTA) was unable to compete with the wild type labelled probe, suggesting that ELT-1 protein interacts directly with GATA site B.

Other *C. elegans* members of the GATA family of transcription factors were tested for their ability to bind the *bro-1* CNE using the same band shift assay; all failed to shift the labelled probe (Figure 3C). Taken together, these data suggest that amongst the members of the GATA family in *C. elegans*, ELT-1 alone mediates *bro-1* transcriptional activity in the seam by direct binding to GATA site B within the CNE.

### ELT-1 regulates *bro-1* expression *in vivo*

The fact that *bro-1* seam expression disappears when ELT-1 binding site B within the *bro-1* regulatory region is deleted suggests that ELT-1 is an activator of *bro-1* expression. To confirm this, we monitored *bro-1::gfp* expression in animals subjected to *elt-1* RNAi and observed a decrease in signal intensity (data not shown). It was necessary to reduce the level of *elt-1* expression by RNAi as *elt-1* alleles are embryonic lethal; therefore only the effects of a small reduction in *elt-1* expression could be analysed. To quantify this, we measured endogenous *elt-1* and *bro-1* transcript levels by qRT-PCR in animals surviving the *elt-1* RNAi treatment. These animals (most of which exhibited seam defects) were found to have a 1.5 fold reduction in *elt-1* transcript levels and a 1.7 fold decrease in *bro-1* transcript levels, demonstrating that ELT-1 plays a major role in regulating *bro-1* expression *in vivo*.

### ELT-1-deficient worms, like *bro-1* mutants, exhibit division failures in the seam lineage

ELT-1 has previously been reported to be essential for seam differentiation and maintenance, with *elt-1(RNAi)* animals having fewer seam cells as assayed by the seam cell marker *scm::gfp* [27]. Significantly, the average number of seam cells in *elt-1(RNAi)* animals (13.6 seam cells per side, ±0.3, n = 83) is very similar to that of *bro-1* mutants (14.0 seam cells per side, ±0.3, n = 91) and significantly lower than animals fed control HT115 bacteria containing the empty feeding vector L4440 (15.8 seam cells per side, ±0.1, n = 30). The cellular basis of this phenotype has not been previously described, therefore lineage analysis was performed to elucidate the cellular mechanism of seam cell loss.

**Figure 4:**
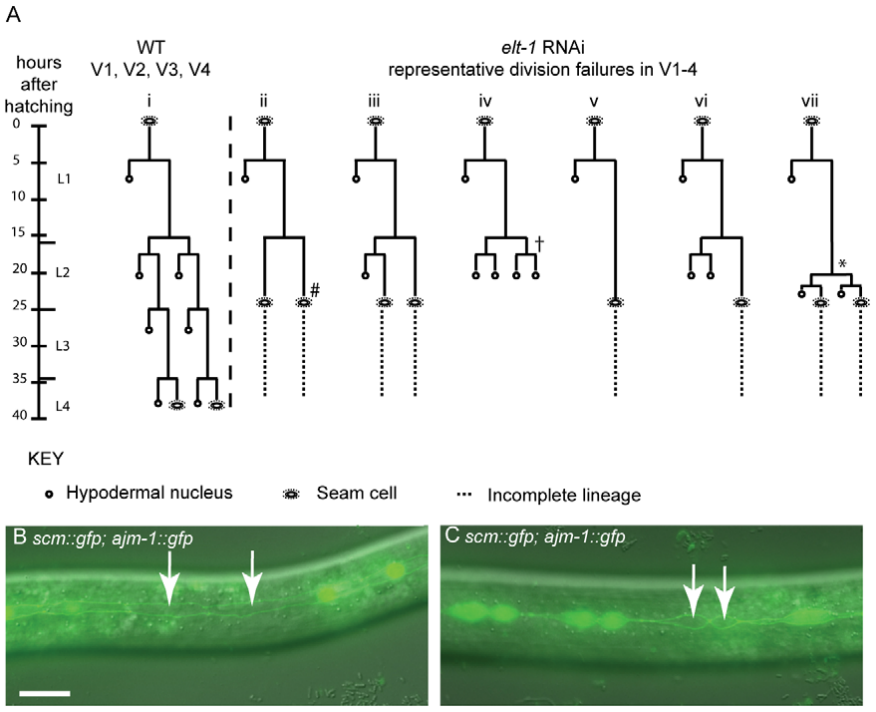
Reduction of *elt-1* function causes V lineage division defects. (A) Lineage traces of WT and *elt-1(RNAi)* hermaphrodites. In *elt-1(RNAi)* worms, seam cells often fail to divide but remain intact and retain their stereotypical seam cell ‘eye’ shape. One or more divisions may be missed, for example in lineage trace ii (#). There was no obvious bias for the V cell involved. Alternatively, certain V lineage seam cells sometimes undergo the symmetrical and asymmetrical divisions during L2, but significantly later relative to both other V cells and to the time of moulting. For example, whereas Vn.p cells should divide first symmetrically and then asymmetrically at the start of L2, these divisions have been observed to occur just before the L3 moult in *elt-1(RNAi)* animals, around 8 hours after they should have occurred (* in lineage trace vii). Similarly, the L2 symmetric and asymmetric divisions in V1-4 should take place slightly ahead of the comparable V6 divisions in WT worms. *elt-1* RNAi, however, often delays these divisions such that they occur after V6p has divided symmetrically and then asymmetrically in L2. In addition to these proliferation defects, aberrant fate changes at division were also occasionally observed (approximately 10% of defects observed); instead of the division producing two seam cells or one seam daughter and one hypodermal daughter, both cells produced adopted the hypodermal fate, becoming smaller and rounder than normal seam cells, losing expression of the *scm::gfp* marker and failing to divide further (e.g. † marked on trace iv). This defect was also observed in *rnt-1* and *bro-1* animals [10]. Designation of the seam fate was based on cell shape, position and division potential, where possible, not *scm::gfp* expression. In no worms were L1 division defects observed. (B) and (C) *elt-1(RNAi)* animals carrying the *scm::gfp* and *ajm-1::gfp* transgenes. Seam cells often lose *scm::gfp* expression (white arrows). 52% of *elt-1* (RNAi) worms were found to have one or more cells bounded by AJM-1::GFP but lacking SCM::GFP (n = 61) compared to 5% in control worms, fed on HT115 bacteria containing the empty L4440 RNAi feeding vector (n = 62). As can be seen, these cells retain their AJM-1::GFP boundary and seam morphology and were not observed to fuse with hyp7. The loss of SCM::GFP appears to be independent of the cells' stage in the cell cycle and can happen before (B) or after (C) division. Scale bar, 25 µm.

*elt-1(RNAi)* worms have variable seam division failures (Figure 4A). These defects were observed during both the symmetrical and asymmetrical divisions of L2, suggesting that the role of ELT-1 is not limited to one type of division. However, given the difficulties in pursuing the lineage analysis past L3 (these worms are very sick), we cannot exclude the possibility that the role of ELT-1 in regulating seam cell division is limited to the L2 stage. Division failures were not observed during the L1 asymmetric division, although embryonic developmental abnormalities often meant that the number of seam cells present at the start of L1 was lower than that of wild type worms. Nevertheless, the ability of the remaining seam cells to undergo the asymmetric L1 division parallels the situation in *rnt-1* and *bro-1* mutants, where division failures are restricted to the subsequent divisions. These division defects are distinct from the classic retarded heterochronic phenotypes, in which stage-specific stereotypical division “cassettes” occur at the wrong stage. In the case of *lin-4*, the L1 pattern of division is re-iterated at later stages, resulting in fewer seam cells overall [28]. In the case of *elt-1(RNAi)* and *rnt-1/bro-1* mutants, the defects seen were not representative of stereotypical division patterns occurring at the wrong stage, but rather involved outright division failures, or occasionally symmetrisation towards the hypodermal fate.

To assess whether the division failures observed in *elt-1(RNAi)* worms were correlated with loss of seam fate, the integrated *scm::gfp* reporter *wIs51* [29] was used as a seam cell marker. We observed that even worms which do not show gross morphological abnormalities, and which therefore can be followed by lineage analysis throughout development, frequently exhibit loss of *scm::gfp* from usually one or two seam cells from the late L1 stage onwards. While the seam cell remains clearly visible under DIC (retaining its distinctive eye-shaped seam morphology), GFP expression fades over 30–60 minutes until the cell shows no fluorescence at all (Figure 4B). To confirm the identity of these ‘seam’ cells that fail to express *scm::gfp*, cell boundaries were visualised using the *ajm-1::gfp* reporter (which marks the apical junctions between seam cells and the surrounding hyp7 syncytium, in which it is not expressed). These cells were always bounded by AJM-1::GFP, confirming their seam identity (as suggested by their morphology). Thus, when seam cells are counted in *elt-1(RNAi)* animals using *scm::gfp*, not all of the seam cells present will be observed. In addition to division failures, therefore, the loss of expression of the ‘seam’ marker *scm::gfp* also likely accounts for the apparent progressive loss of seam cells previously reported in *elt-1(RNAi)* worms [27].

Interestingly, a similar phenotype is observed in *bro-1* and *rnt-1* mutants (data not shown). However, while loss of this marker has been attributed to degeneration of seam cells [27], we have not observed this phenomenon in either *rnt-1*/*bro-1* mutants or *elt-1(RNAi)* worms; seam cells retain their characteristic “eye” shape and position within the seam line, but may lose *scm::gfp* in these backgrounds. Given the prevalence of using *scm::gfp* as a marker of seam fate, we were surprised to observe that *scm::*gfp expression does not in fact seem to be tightly correlated with proliferative potential; lineage analysis revealed that cells that stop expressing *scm::gfp* often undergo the correct number of divisions at the appropriate times. Conversely, we observed division failures in seam cells strongly expressing *scm::gfp*. Taken together, this suggests that while *scm::gfp* may mark seam cells during normal development, it is not an infallible marker for seam cell fate, perhaps because particular elements of seam ‘fate’ are uncoupled in *elt-1(RNAi)* and *rnt-1/bro-1* mutant animals.

Cells that remained in the seam line but failed to divide, always retained expression of *ajm-1::gfp*, regardless of whether or not they expressed *scm::gfp* (Figure 4B and 4C). This suggests that their failure to divide results not from a change of fate *per se* (from seam to hypodermis), but rather from a loss of proliferative ability. This is supported by *dpy-7p::yfp* expression, which marks cells that have adopted a hypodermal fate but is not expressed in seam cells; cells bounded by AJM-1::GFP never expressed *dpy-7p::yfp*, even when they fail to undergo scheduled divisions (data not shown).

### ELT-1 works independently from BRO-1 to prevent seam cells fusing inappropriately with the hypodermis

**Figure 5:**
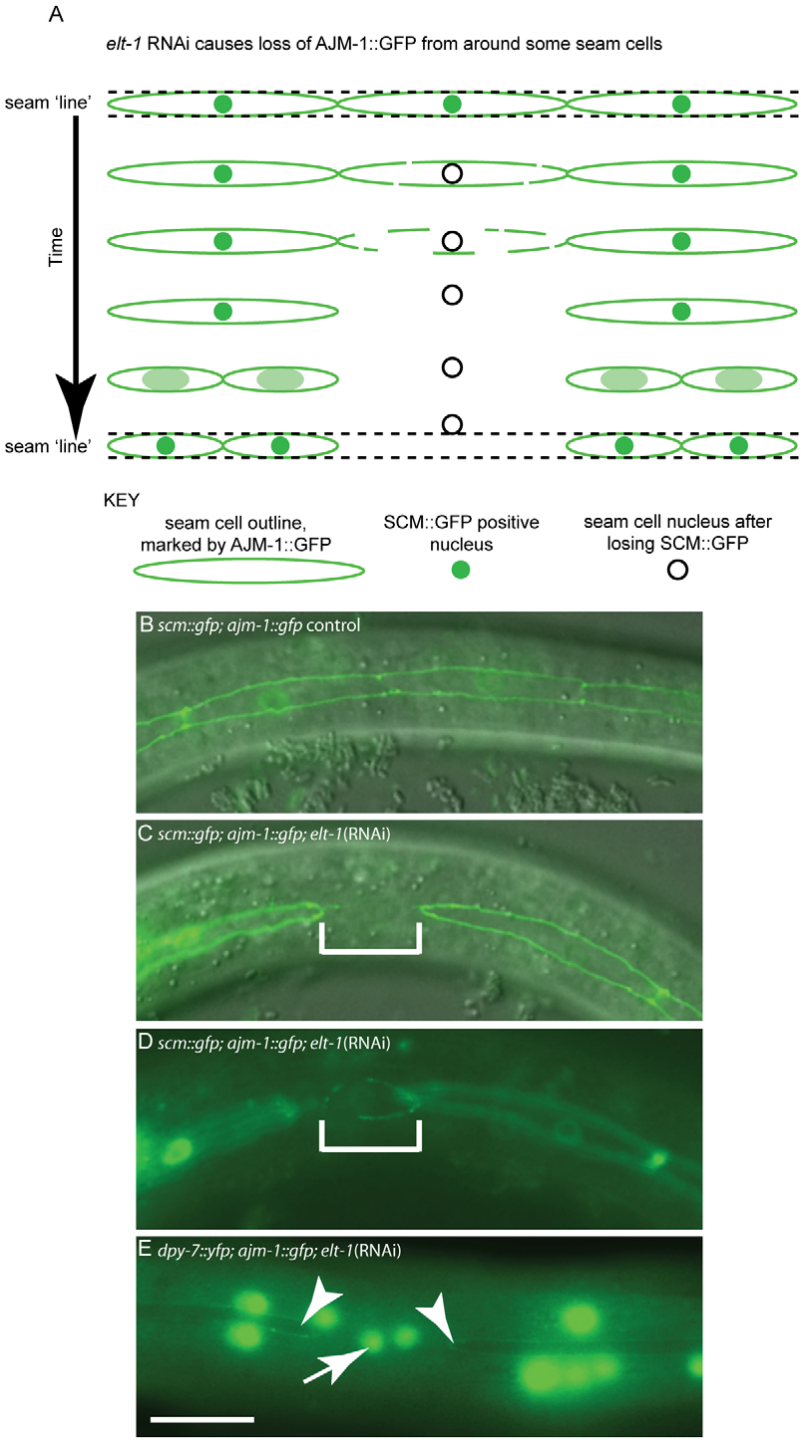
*elt-1* RNAi causes division-independent seam to hypodermis fate transitions. In addition to division defects, loss of seam fate was also observed between cell divisions following *elt-1* knockdown. (A) Schematic diagram of AJM-1 loss from one cell in the V lineage, involving the gradual loss of AJM-1::GFP from around the cell until the cell is completely unbounded. SCM::GFP expression is always absent in such cells. Over a period of several hours, the cell becomes smaller and rounder and moves out of the line of the seam, into the hyp7 syncytium. Subsequently, these cells fail to divide, in contrast to cells that remain in the seam lineage and thus retain their stem fate. (B) Wild type seam lineage in animals expressing both *ajm-1::gfp* and *scm::gfp*, showing cells in a continuous line. (C) and (D) show the same cell in two different planes in an *elt-1*(RNAi) animal in which seam cells lose their AJM-1::GFP boundary (representative cell shown in bracketed region), changing shape and plane as they move into the hyp7 syncytium (change of shape and plane obvious in D). In order to quantify inappropriate fusion in these worms we scored animals with one or more breaks in the seam line. In *elt-1(RNAi)* animals 68% of animals had breaks in the seam (n = 44). In control animals (exposed to HT115 bacteria containing the empty RNAi feeding vector L4440) 1.8% of animals had breaks (n = 56). (E) Using worms expressing both AJM-1::GFP and *dpy-7p*::yfp, it is possible to show that cells which lose their AJM-1 boundary and move out of the seam lineage as a result of *elt-1* RNAi, subsequently switch on *dpy-7* expression (indicated by white arrow), a marker of hypodermal fate. Adjacent seam cells which retain AJM-1::GFP expression show no *dpy-7* expression (white arrow heads).

Unlike the situation in *bro-1* mutants, however, observation of *elt-1(RNAi)* animals carrying the integrated *ajm-1::gfp* construct revealed that some cells, at the same time as losing *scm::gfp* expression, lose AJM-1::GFP, used here as a marker of seam cell boundaries (data not shown and Figure 5A–5D). This may be predictive of inappropriate fusion with the hyp7 syncytium. Cells were never observed that lost *ajm-1::gfp* but retained *scm::gfp*. The ‘disintegration’ of the AJM-1::GFP continues until only vestigial traces are evident between the two seam cells on either side of the affected cell, as shown in Figure 5D.

In the hours subsequent to losing *ajm-1::gfp* expression, we observed that the seam cell tends to become rounder and move out of the line of the seam, with both the morphological and positional changes being indicative of the acquisition of hypodermal fate. To test this, a strain carrying the integrated transgenes *ajm-1::gfp* and *dpy-7::yfp* was used. In several cases, cells were observed during lineage analysis losing *ajm-1::gfp* and then acquiring *dpy-7::yfp* expression. Gaps in *ajm-1::gfp* expression in the seam were correlated with the presence of *dpy-7::yfp*-expressing nuclei which had not yet, or only partially, moved out of the line of the seam (Figure 5E). Thus, *elt-1* RNAi causes an additional phenotype not observed in *rnt-1* or *bro-1* mutants, whereby some seam cells differentiate inappropriately by fusing with the hypodermal syncytium.

Overall, our data suggests three distinct phenotypes observed in *elt-1(RNAi)* animals. The first, in common with *rnt-1* and *bro-1* mutants, involves division failure in the absence of a permanent change in cell fate (i.e. not involving fusion with the hypodermis). Secondly, loss of SCM::GFP is also observed in *elt-1(RNAi)* animals (as well as in *rnt-1* and *bro-1* mutants), but we found this to be independent of division failure. Thirdly, in *elt-1(RNAi)* animals but not in *rnt-1/bro-1* mutants, we observed inappropriate adoption of the hypodermal fate as shown by the acquisition of DPY-7::YFP, preceded by loss of *scm::gfp* expression and of AJM-1::GFP from the apical junctions. While these cells always lose SCM::GFP, fusion with the hypodermis is not always the consequence of SCM::GFP loss. However, AJM-1::GFP is very tightly linked to the seam fate; loss of AJM-1::GFP is always coupled with fusion to the hyp7 syncytium and acquisition of the hypodermal fate.

### The *elt-1* (RNAi) fusion defect is dependent on EFF-1

**Figure 6:**
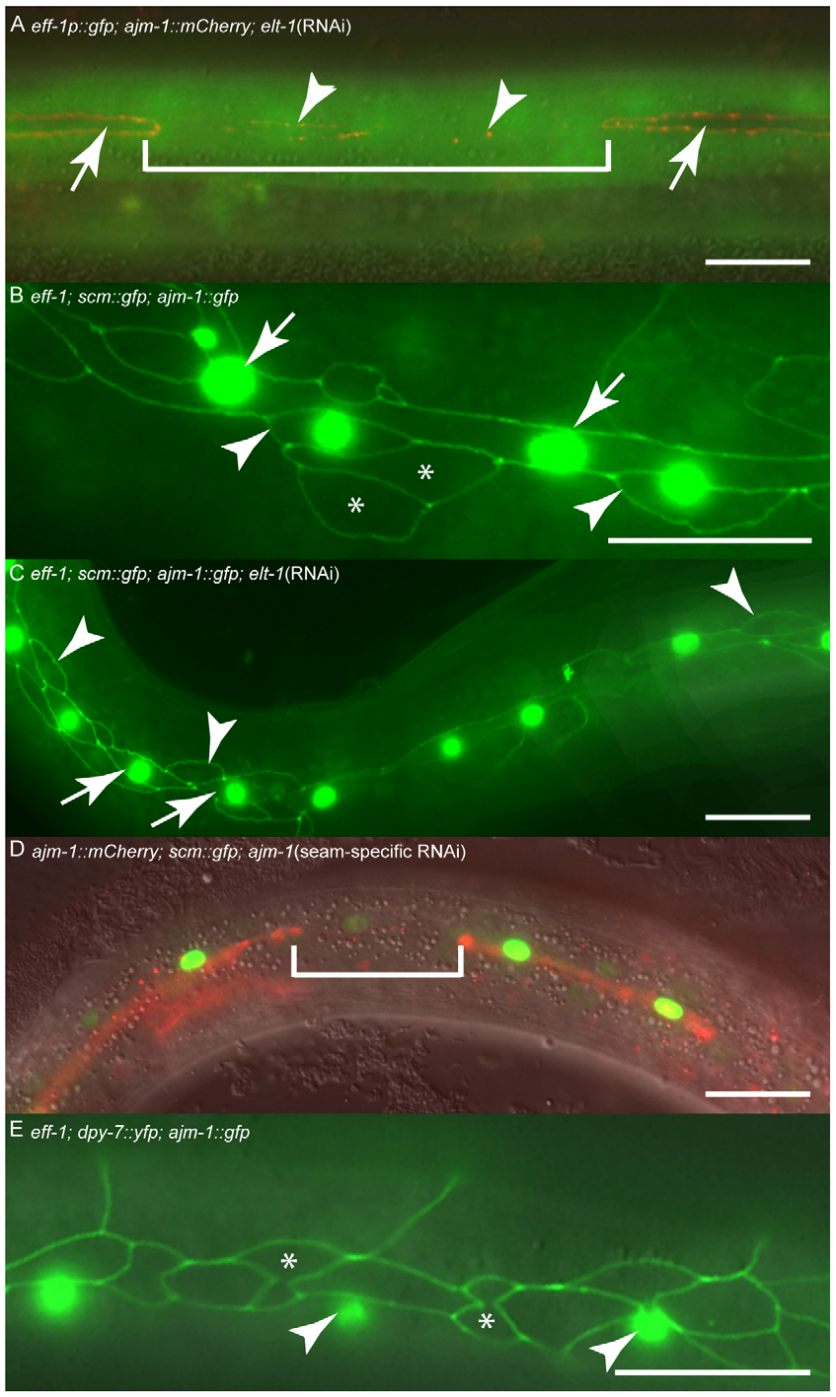
ELT-1 represses *eff-1* expression in seam cells to preserve stem cell-like identity and maintain apical junction integrity. (A) Hermaphrodite carrying *eff-1p::gfp* and *ajm-1::mCherry* transgenes, subjected to *elt-1* RNAi. Within the bracketed region a seam cell is in the process of losing *ajm-1* expression, and only has vestiges of AJM-1::GFP remaining (arrowheads). This cell is now expressing *eff-1p::gfp*. In contrast, cells retaining their AJM-1 border never express *eff-1p::gfp* (white arrows). (B) *eff-1* mutant hermaphrodite expressing *scm::gfp* and *ajm-1::gfp*. *scm::gfp* expression in the “true” seam is shown with white arrows. Anterior daughters of seam divisions sometimes retain expression of *scm::gfp* (white arrowheads), particularly where they remain in contact with the true seam (presumably these are the most recent daughters). Other anterior daughters do not express *scm::gfp* but retain their AJM-1 boundary (asterisks). In order to quantify inappropriate fusion in these worms we scored animals with one or more breaks in the seam line. In *eff-1* mutants 3% of animals had breaks in the seam (n = 31). (C) *eff-1* mutant hermaphrodite expressing *scm::gfp* and *ajm-1::gfp*, subjected to *elt-1* RNAi. In these animals, no AJM-1 breakdown is observed, either around seam cells (white arrows) or around anterior daughters of asymmetric seam divisions (white arrowheads), demonstrating that the *elt-1* (RNAi) induced fusion of seam cells with hyp7 is suppressed in *eff-1* mutants. In order to quantify inappropriate fusion in these worms we scored animals with one or more breaks in the seam line. In *eff-1* mutants subjected to *elt-1* RNAi 0% of animals had breaks in the seam (n = 20), compared with 68% in *elt-1(RNAi)* animals alone (n = 44, Figure 5A, 5C, 5D). (D) Seam-specific RNAi strain hermaphrodite subjected to RNAi of *ajm-1*. In this experiment the RNAi deficient mutant *rde-1* was used, rescued with *rde-1* cDNA expressed under the control of the seam-specific SCM promoter, as described in detail in the Text S1. The bracketed cell has lost its AJM-1 border and the SCM::GFP has become much fainter. (E) *eff-1* hermaphrodite carrying *dpy-7p::yfp* and *ajm-1::gfp* transgenes. Anterior daughters of seam divisions retain their AJM-1 borders but never express *dpy-7* (asterisks). *dpy-7* expression is only observed in the syncytial hypodermis surrounding these cells (white arrowheads). Scale bar, 25 µm.

If cells are lost from the seam in *elt-1(RNAi)* animals because of inappropriate fusion with the hyp7 syncytium, we would anticipate that ectopic *eff-1* expression would be evident, as the fusogen EFF-1 is known to be required for this fusion event [30]. To test this, a transgenic strain carrying both an *eff-1* transcriptional reporter, which normally expresses in dorsal and ventral hypodermis but not in seam cells, and *ajm-1::mCherry* was used. In *elt-1(RNAi)* animals, we observed ectopic *eff-1p::gfp* expression in seam cells that had lost their *AJM-1* boundary (Figure 6A). Furthermore, we found that *elt-1* RNAi induced fusion of seam cells with the hyp7 syncytium was suppressed in *eff-1* mutants (Figure 5A, 5C, 5D and Figure 6C). Taken together, these data suggest that ELT-1 represses *eff-1* expression in seam cells in order to prevent fusion of these cells with the surrounding hypodermal syncytium, thus maintaining their distinct stem cell-like fate.

### Seam cell membrane integrity, as marked by apical junctions, is required to maintain stem cell-like identity and sufficient to prevent differentiation

In order to address more directly the role of seam cell boundaries in determining the stem cell-like identity of seam cells, we knocked down the expression of components of apical junctions by RNAi. Given the essential functions of these proteins in a variety of cell types throughout development, it was necessary to use a seam-specific RNAi approach [31]. Knockdown of either *ajm-1*, *let-413* or *dlg-1* gave a similar phenotype, involving breaks in the AJM-1::GFP boundary, associated loss of *scm::gfp* expression and withdrawal from further division (Figure 6D; *let-413* and *dlg-1* data not shown). When *ajm-1* was knocked down by RNAi, 66% of animals displayed breaks in the seam boundary as marked by *ajm-1::mCherry* (n = 50), whereas in controls not subjected to RNAi 5% of animals displayed breaks (n = 36) (the odd break in the seam would be expected due to mosaicism of the *ajm-1::mCherry scm::gfp* array). Therefore, seam cell membrane integrity, as marked by apical junctions, is essential for the maintenance of seam stem cell-like fate.

Next, we wanted to examine whether the presence of an intact boundary around the seam cells is sufficient to specify seam fate. This could happen in two ways: the boundary could either promote seam fate, or block differentiation signals from the surrounding environment. To test this, we used an *eff-1* mutant in which anterior daughters retain their AJM-1::GFP marked boundary and remain in contact with the seam line instead of moving into the hypodermis. In other words, ectopic AJM-1::GFP boundaries are present in this strain. To assess whether these cells inappropriately retain the seam fate we monitored *scm::gfp* expression, as well as *dpy-7::yfp* expression. Firstly, we found that ectopic AJM-1::GFP bordered cells frequently do not express *scm::gfp* (Figure 6B), suggesting that these cells do not retain all aspects of seam fate. Perhaps this is not surprising, given that these cells are the anterior daughters of asymmetric seam divisions and would have already received the instruction to withdraw from further proliferation. However, we never observed *dpy-7::yfp* expression in these cells (Figure 6E), indicating that differentiation is repressed. This suggests that fusion of seam cells with hyp7 (mediated by EFF-1) is essential as a trigger for differentiation in this context. These cells are therefore in developmental ‘limbo’, being neither completely seam nor hypodermis. Thus, although the anterior daughters of asymmetric seam divisions are destined to differentiate at division, the nature of the differentiation event is not specified at this stage and requires further inputs; it is not until cell fusion and membrane breakdown occurs, as marked by the loss of apical junction boundaries, that the hypodermal fate is adopted.

**Figure 7:**
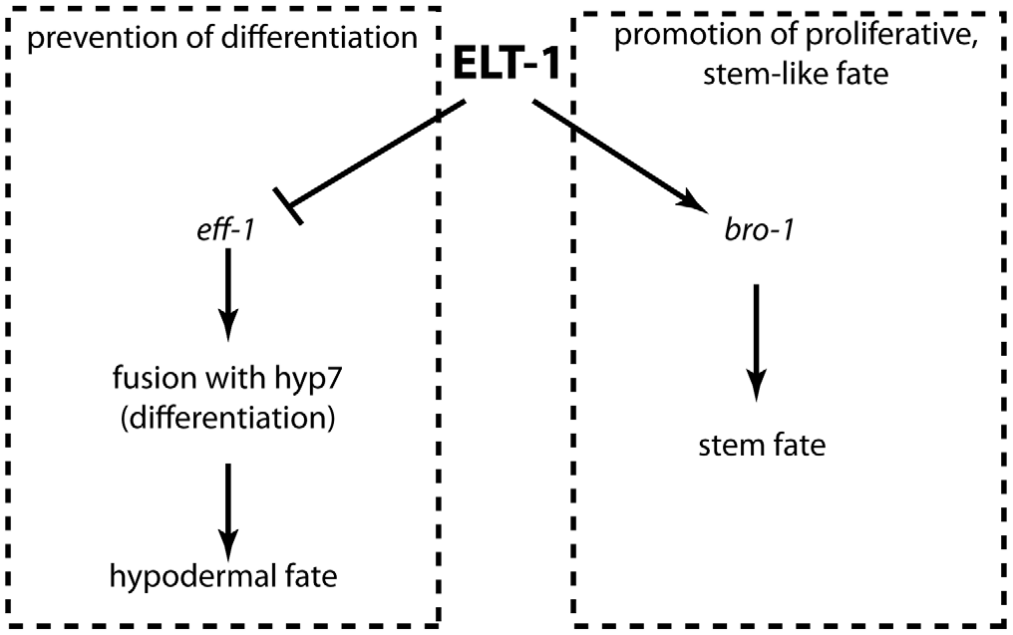
ELT-1 maintains the seam stem cell-like fate by promoting cell proliferation and preventing inappropriate differentiation. Model to illustrate the relationship between ELT-1, BRO-1 and EFF-1 in the maintenance of the seam stem cell-like fate. Direct transcriptional activation of *bro-1* by ELT-1 promotes proliferation, while repression of *eff-1* by ELT-1 (either directly or indirectly, for example through *elt-5/6*) represses fusion mediated by EFF-1, thereby preventing differentiation.

Overall, therefore, seam cell boundaries may not be sufficient to specify all aspects of seam fate, but they are sufficient to prevent differentiation. Our data therefore suggest that the seam stem cell-like fate is retained by the blocking of “hypodermalizing” signals in cells that are bounded by apical junctions. ELT-1, therefore, plays dual roles in specifying the stem cell-like properties of the seam cells; on the one hand activating proliferative potential *via bro-1*, and on the other preventing inappropriate differentiation through the repression of *eff-1* and consequent maintenance of seam boundaries (Figure 7).

## Discussion

*C. elegans* seam cells are an excellent model for stem cell-like modes of division, sitting as they do at the crossroads between the developmental decision to proliferate (thus renewing the pool of pluripotent precursors), or to pursue a differentiation pathway towards one or more specialised cell types. We used comparative genomics coupled with a yeast one-hybrid screen and promoter deletion analysis to define upstream regulators of *bro-1* expression; a gene known to be essential for seam cell proliferation. Surprisingly, we found that sequences necessary and sufficient for the expression of *bro-1* lie exclusively within an intron. Our evidence adds strength to the concept that highly conserved non-coding sequences correlate with biologically important enhancer elements [32], [33]. Multiple lines of evidence suggest that ELT-1 binds to this intronic sequence; in a yeast one-hybrid screen using the CNE as bait, ELT-1 was identified in 92% of positive clones, whilst an EMSA band shift experiment not only confirmed this finding, but demonstrated that the putative GATA site towards the 3′ end of the CNE (GATA site B) is essential for ELT-1 binding, an observation confirmed by rescue experiments.

Taken together, evidence from our yeast one-hybrid screen, *in vitro* binding assay, rescue experiments and quantitative RT-PCR experiments suggest that ELT-1 is a direct upstream transcriptional regulator of *bro-1*. This model is supported by the similarities between *elt-1* (RNAi) and *bro-1* mutant phenotypes in terms of failures of seam cell divisions, and loss of *scm::gfp* expression. It therefore seems highly likely that *elt-1* and *bro-1* (as well as *rnt-1*) function within the same developmental pathway to promote seam cell proliferation. It is interesting to note that the L1 (asymmetric) division is robust and appears unaffected in both *elt-1* RNAi and *bro-1/rnt-1* mutant worms. This division is most likely regulated by a separate pathway. The involvement of both the CBFβ/RUNX complex and a GATA transcription factor in the regulation of the proliferation of the stem cell-like seam cells is reminiscent of the situation in *Drosophila* and in mammals, where GATA and RUNX factors work together to regulate blood cell formation [34]. Indeed, mammalian *Runx1* is known to be transcriptionally activated by Gata2 [22]. Thus, the direct regulatory relationship between ELT-1 and *bro-1* suggests yet another mode of interaction between members of these two gene families.

*elt-1(RNAi)* worms display a striking phenotype which is not observed in *bro-1* mutant animals, however. This involves loss of the apical cell junction marker *ajm-1::gfp* from some seam cells, followed by movement of the cell out of the seam and subsequent differentiation into the hypodermal fate. It has previously been argued that seam cell phenotypes (loss of *scm::gfp* positive cells and gaps in *ajm-1::gfp* expression) in *elt-1(RNAi)* worms do not result from inappropriate fusion of the seam with the hypodermis, on the basis that *scm::gfp*-expressing cells are never observed in the hypodermal syncytium [27]. However, here we argue that cells do indeed fuse with hyp7 in *elt-1(RNAi)* animals, but first lose their *scm::gfp* and AJM-1 boundary, as well as changing their morphology as they undergo the transition from seam to hypodermal fate and begin to express hypodermal markers. Thus, we suggest that the breaks in AJM-1::GFP result not from degeneration of the seam cells but are the result of a transition between two cell fates; the proliferative seam fate, which is associated with the AJM-1::GFP boundary, and the differentiated syncytial hyp7 fate, which is not.

In terms of its *bro-1*-independent role in the seam, *elt-1* appears to function upstream of EFF-1. The role of the fusogen *eff-1* is critical in the seam [30] and is responsible for promoting the fusion of hypodermal seam daughters with the hyp7 syncytium by causing the formation of pores in the membrane [35]. Indeed, *eff-1* over-expression has been shown to result in inappropriate fusion of seam cells with hyp7 [36]. This phenotype is strikingly similar to that seen in *elt-1(RNAi)* animals, and suggests that ELT-1 acts to repress *eff-1* in the seam, thereby preventing seam cells from fusing with hyp7. Our finding that ectopic *eff-1* expression is observed in *elt-1(RNAi)* animals confirms this.

Inappropriate fusion of the seam cells with the hypodermis has been reported previously; this phenomenon has been observed in embryos and newly hatched animals in which *elt-5* and *elt-6* (which act redundantly) had been knocked down by RNAi [29]. Moreover, similar to the observations described here for *elt-1(RNAi)* animals, fusion in the case of *elt-5/elt-6* RNAi was accompanied by gradual dissolution of the AJM-1::GFP boundary around the seam cells [29]. We therefore suggest that there is a network of GATA factors acting to prevent inappropriate differentiation of seam cells throughout development. In addition, other transcription factors have been shown to regulate seam cell development, for example NHR-25, BAF-1 and CEH-16 as well as heterochronic regulators like LIN-14 and LIN-29 [14], [15], [28], [37]–[39]. The interactions between all these genes will be an interesting area for future study.

The apparent close relationship between the presence of intact apical junctions and the stem cell-like properties of seam cells is suggestive of similarities with stem cells in *Drosophila*. In the testes and ovaries of *Drosophila*, germline stem cells (GSCs) are retained in what has been termed a niche; the niche concept, introduced over 30 years ago [40], describes how stem cells can be maintained in a proliferative state by signals from a microenvironment, consisting of cells and the extracellular components they produce. The niche is essential for the stem properties of such cells and, as these cells move out of the niche, so they lose these properties in favour of differentiated cell fates. In this way, far from merely creating an inert environment in which the *Drosophila* GSCs reside, the cells around these stem cells provide cues which regulate the maintenance of the stem cell pool, physically anchor the GSCs to the niche, and even control the polarity of the stem cells, determining the positions of the daughters of GSC divisions relative to one another and to the niche. The mechanisms underlying the complex interactions between GSCs and their niche microenvironment involves extracellular signalling [41]–[45] as well as physical adhesion of stem cells to the niche [46], with the DE-cadherin and Armadillo/β-catenin apical junction complex being both important in recruiting GSCs to the niche and required for the maintenance of the stem cell pool. Loss of either of these proteins results in dramatic depletion of GSCs from the niche. Here, we show that *C. elegans* apical junction proteins are required to maintain the undifferentiated stem cell-like fate of the seam cells.

Perhaps there is something analogous to a seam stem cell “niche” in *C. elegans*, in the sense that cell contacts (marked by, and dependent on, apical junction proteins) are required to maintain a microenvironment in which the seam cells are prevented from differentiation. The importance of cellular contacts for seam development has previously been recognized. For example, the developmental fate of the V5 seam cell has been shown to be dependent on correct seam cell contacts either side [47], and proliferation of the seam cells has been shown to be perturbed when contacts between seam cells are not properly re-established following division [15]. Thus, both the proliferation and differentiation of the seam cells has been shown to be dependent on signals from the surrounding microenvironment, raising the question of whether they do in fact reside in a niche. In support of the niche concept, we find that cells that have withdrawn from the proliferation programme as a result of asymmetric division, but which have failed to fuse with the hyp7 syncytium (as a result of *eff-1* mutation), do not express markers of differentiation like *dpy-7*. In other words, the boundary provides protection from differentiation signals. However, this notion of a niche has to remain speculative in the absence of defining the nature of these signals.

Intriguingly, the regulation we have discussed in seam cells, in which ELT-1 represses *eff-1* in order to prevent fusion and differentiation of cells that have the proliferative fate, mirrors the situation in vulval precursor cells (VPCs). In the developing vulva, the 6 VPCs P3.p–P8.p are prevented from fusing with the hyp7 syncytium in early larval stages (in L3 this exclusion from hyp7 is limited to P5.p–P7.p) [48]. Fusion with hyp7 acts to limit the developmental potential of Pn.p cells (as it does with anterior seam daughters) and is restricted to those that flank the developing vulva [48]. LIN-39 acts to prevent this fusion by repressing *eff-1* in P3.p–P8.p during L1 and in P5.p–P7.p during L3 [49]–[51]. In *eff-1* mutants, unfused VPCs fail to differentiate into the hypodermal fate, retaining their AJM-1 boundary and at least some aspects of the vulval fate. These cells fail to proliferate, however, suggesting that other signals are required for the induction of normal vulval development. This is analogous to the situation with unfused seam cells in *eff-1* mutants, which also fail to divide once they leave the seam line, remaining in developmental “limbo”. In both cases, however, fusion with hyp7 and associated breakdown of cell boundaries is required for cells to take on the differentiated hypodermal fate. In both the seam and the developing vulva, therefore, only those cells that are prevented from fusing with hyp7 (thereby retaining their boundaries) are protected from differentiation and retain further developmental and proliferative potential.

Overall, we present a model (Figure 7) in which the GATA factor ELT-1 plays important dual roles in maintaining the ‘stemness’ of the seam cells, by both promoting the proliferative fate and preventing differentiation. Firstly, ELT-1 acts directly through *bro-1* to promote proliferation and self-renewal of the seam. Secondly, ELT-1 is essential for maintaining the integrity of the seam cell compartment, as marked by apical junctions. In fulfilling this latter role, ELT-1 works through EFF-1. When *eff-1* is repressed, the boundaries around the seam cells are maintained, and thus differentiation is prevented. We also find that apical junction components themselves are important for maintaining seam cell fate, but are not arguing that ELT-1 acts directly on apical junction components. Indeed, as has been previously suggested, apical junction breakdown could be a relatively late event in the cell fusion process [35], [52]. Taken together, these data suggest that the seam cells reside in a microenvironment in which they are protected from differentiation by the boundary that separates them from the hyp7 syncytium. Thus, the seam microenvironment may satisfy the criteria of a niche in certain respects, protecting seam cells from influences that would otherwise trigger differentiation. The GATA factor ELT-1 works through *bro-1* to promote seam cell proliferation and through *eff-1* to maintain seam cells in the undifferentiated state.

## Materials and Methods

### Strains and maintenance of worms

All strains used were derived from the wild type N2 Bristol strain. Manipulations and maintenance of strains were performed as previously described [53]. Strains used are described in Table S1.

### Lineage analysis and microscopy

Lineage analysis was performed as previously described [12]. For lineage analysis of *elt-1(RNAi)* animals, 3 µl of a freshly prepared solution of M9 and *HT115 E. coli* cells expressing the *elt-1* dsRNA (scraped from a 2 mM IPTG NGM plate, on which they had been growing at 20°C for several days) was placed on the pad. For each slide, a single worm was transferred into this drop with an eyelash pick. A coverslip was then slowly lowered on top of the worm, and microscopy performed with Nomarski (DIC) optics and a 100× oil immersion objective (Zeiss). Photomicrographs were taken using a 63× Zeiss oil immersion objective and Axiovision software (Release 4.5).

### RNAi

*elt-1* knockdown by RNAi was performed as described previously [27], using an identical feeding construct to *pPM88*, named *pAW565*. The seam specific RNAi strategy is described in Text S1 and all other RNAi was performed by feeding as previously described [54].

### Plasmid construction

Plasmids used in transgenic strains are described in Table S1 and detailed cloning strategies described in Text S1.

### Construction of transgenic worms

Injections were performed as described previously [55] using the *unc-119* ^+^ (*pDP#MM016β*) transformation marker [56]. Constructs were injected at 10–20 ng/µl.

### Band shift experiments

cDNAs of *elt-1*, *elt-2*, *elt-3*, *elt-5* and *elt-6* were amplified from a mixed stage cDNA preparation using Phusion polymerase (Finnzymes). The PCR products were cloned into the *pCR*®*-XL-TOPO* vector (Invitrogen) and the *TNT®* Quick Coupled Transcription/Translation kit (Promega) was then used for *in vitro* transcription and translation. To make the labelled probes, oligonucleotides covering ‘GATA site B’ were synthesised (WT probes: *CB204* gatccgacaagattacaatccacat, *CB206* atgtggattgtaatcttgtcggatc; mutant probes: *CB205* gatccgacaatagtacaatccacat; *CB207* atgtggattgtactattgtcggatc), annealed by heating and gradual cooling, and labelled with [γ-^32^P] dATP (for hot WT probes) or without [γ -^32^P] dATP (for cold, competitor probes). The DNA binding reaction was carried out on ice for 30 minutes before the reaction mixture was loaded onto a 7% non-denaturing polyacrylamide gel and run at 4°C in 0.5×TBE.

### Yeast one-hybrid screen

The entire 122 bp *bro-1* CNE was used as bait in the yeast one-hybrid screen; three copies were inserted in the forward direction into the Clontech Matchmaker vectors *pHisi-1* and *pLacZi* and integrated into yeast strain YM4271 [57] as described in Text S1. *YM4271* [p*bro-1*HIS; p*bro-1*LAC] was then transformed with a mixed stage transcription factor cDNA library and plated onto –his, -leu, -ura 15 mM 3-AT SD plates. All colonies that grew within 3 days were assayed for lacZ expression [58], and after re-isolating plasmids and re-checking, positive clones were sequenced.

### Quantitative RT-PCR analysis

qRT-PCR was performed on synchronised worms obtained by bleaching gravid animals and seeding the eggs onto *elt-1* RNAi or L4440 control plates. Larvae were harvested after 1–2 days by washing with M9. RNA was extracted by the hot phenol method [59] and mRNA levels of *elt-1/bro-1* and a normaliser (*nuo-2*, expressed in all seam cells) were assessed using SYBR-Green and a Qiagen Rotor-Gene Q machine. Expression levels were assayed by the 2^−ΔΔC^T method [60].

## Supporting Information

Table S1 Strains used in this study.
(DOC)

Text S1 Experimental protocols used in this study.
(DOC)
