# Supplementary material for: The Caenorhabditis elegans GATA Factor ELT-1 Works through the Cell Proliferation Regulator BRO-1 and the Fusogen EFF-1 to Maintain the Seam Stem-Like Fate
Source: PLoS Genet. 2011 Aug 4;7(8):e1002200. doi: 10.1371/journal.pgen.1002200 (PMC3150447; doi:10.1371/journal.pgen.1002200)
Supplement: Table S1 — Strains used in this study. (DOC) [file pgen.1002200.s001.doc]

| Strain | Genotype | Plasmids in extrachromosomal arrays |
| --- | --- | --- |
| *AW196* | *unc-119(ed3) III; him-8)e1489) IV; ouEx44[pAW304 + pAW302 +unc-119^+^]* | *pAW304 = bro-1CNE::gfp*  *pAW302 = scm::rfp* |
| *AW204* | *bro-1(tm1183) I; unc-119(ed3) III; him-8(e1489) IV; ouEx49[pAW303 + unc-119^+^]* | *pAW303 = bro-1::dsRED2* |
| *AW197* | *ccIs4251[myo-3::gfp] I; unc-119(ed3) III; him-8(e1489) IV; ouEx45[pAW305 + unc-119^+^]* | *pAW305 = bro-1ΔCNE::dsRED2* |
| *AW198* | *bro-1(tm1183) I; unc-119(ed3) III; him-8(e1489) IV; ouEx45[pAW305+ unc-119^+^]* | *pAW305 = bro-1ΔCNE::dsRED2* |
| *AW528* | *bro-1(tm1183) I; unc-119(ed3) III; him-8(e1489) IV; ouEx214[pAW373+ unc-119^+^]* | pAW373 = *bro-1CNE::bro-1cDNA::gfp* |
| *AW313* | *bro-1(tm1183) I; him-8(e1489) IV* |  |
| *CB1489* | *him-8(e1489) IV* |  |
| *AW326* | *bro-1(tm1183) I; unc-119(ed3) III; him-8(e1489) IV; ouEx115[pAW390 + unc-119^+^]* | *pAW390 = bro-1CNE ΔGATA site A::bro-1cDNA::gfp* |
| *AW529* | *bro-1(tm1183) I; unc-119(ed3) III; him-8(e1489) IV; ouEx215[pAW393 + unc-119^+^]* | pAW393*= bro-1CNE ΔGATA site B::bro-1cDNA::gfp* |
| *AW298* | *wIs78 [scm::gfp + ajm-1::gfp + unc-119^+^] IV; him-5(e1490) V* |  |
| *AW525* | *unc-119(ed3) III; syIs78 [ajm-1::gfp + unc-119^+^]; arIs99 [dpy-7p::yfp]* |  |
| *AW560* | *unc-119(ed3) III;ouEx240 [pAW564 + ajm-1::mcherry + unc-119^+^]* | *pAW564 = eff-1p::gfp* |
| *AW551* | *eff-1(hy21) II; wIs78 [scm::gfp + ajm-1::gfp + unc-119^+^]IV* |  |
| *AW534* | *bro-1 (tm1183) I; wIs78 [scm::gfp + ajm-1::gfp + unc-119^+^]IV* |  |
| *AW552* | *unc-119(ed3) III; rde-1(ne219) V; ouEx233 [pMF1 + ajm-1::mcherry + pAW559 + unc-119^+^]* | *pAW559 = scm::rde-1cDNA*  *pMF1 = scm::gfp* |
| *AW536* | *eff-1(hy21) II; arIs99 [dpy-7p::yfp]; syIs78 [ajm-1::gfp + unc-119^+^]* |  |
| *AW186* | *bro-1 (tm1183) I; him-5 (e1490) wIs51 [scm::gfp + unc-119^+^] V* |  |
| *AW60* | *him-5 (e1490) wIs51 [scm::gfp + unc-119^+^] V* |  |
| *AW187* | *rnt-1 (tm388) I; him-5 (e1490) wIs51 [scm::gfp + unc-119^+^] V* |  |
| *AW561* | *unc-119(ed3) III; ouEx241 [pAW545 + unc-119^+^]* | *pAW545 = non-conserved region of intron 2::gfp* |
| *AW562* | *unc-119(ed3) III; ouEx242 [pAW546 + unc-119^+^]* | *pAW546 = intergenic region upstream of* bro-1 *ORF::gfp* |
| *AW527* | *unc-119(ed3) III; ouEx213 [ajm-1::mcherry + pAW549 + unc-119^+^]* | *pAW549 = eff-1p::gfp* |
