# Supplementary material for: The Caenorhabditis elegans GATA Factor ELT-1 Works through the Cell Proliferation Regulator BRO-1 and the Fusogen EFF-1 to Maintain the Seam Stem-Like Fate
Source: PLoS Genet. 2011 Aug 4;7(8):e1002200. doi: 10.1371/journal.pgen.1002200 (PMC3150447; doi:10.1371/journal.pgen.1002200)
Supplement: Text S1 — Experimental protocols used in this study. (DOC) [file pgen.1002200.s002.doc]

**Experimental Protocols S1**

**Seam-specific RNAi**

This was performed essentially as described [31]. *pAW559*, containing *rde-1* cDNA driven by the seam-specific *“scm”* promoter [29], was co-injected into *AW553*, together with *scm::gfp* (*pMF1*), *ajm-1::mCherry* and an *unc-119^+^* rescuing plasmid to make strain *AW552*. *ajm-1* RNAi was performed by injection of dsRNA into *AW552*.

**Plasmid construction**

The *bro-1* CNE minimal promoter construct was made by first amplifying the 122bp region from genomic DNA and cloning the PCR product into the *pCR2.1* vector (TOPO TA cloning kit, Invitrogen), producing *pAW553*. The cloned region was then cut from this construct using *Hind*III and *Xba*I, and inserted into the Fire Lab vector *pPD107.94*, cut with the same restriction endonucleases. This vector contains the *pes-10* minimal promoter sequence and GFP, downstream of the multiple cloning site into which the CNE was inserted. The resulting construct was named *pAW304* (*bro-1* CNE::*GFP*).

*pAW373* (*bro-1* CNE driving *bro-1* cDNA::*gfp*) was made in two steps. Initially, the Fire Lab vector *pPD49.26* was cut with *Hind*III and *Bam*HI. The *bro-1* CNE was then cut from *pAW553* using *HindIII* and *Pst*I and, from *pPD107.94*, the *pes-10* promoter (Fire Lab vector, 1997 Kit) was cut with *Pst*I and *Bam*HI. These three fragments were then ligated together, making *pAW400*. The *bro-1*cDNA::*gfp* PCR product was produced in two stages, by fusion PCR [23]. To amplify *bro-1* cDNA with a 3’GFP tag, the primers *CB164* (atgaaaagaacgacaacggac) and *CB23* (cgacctgcaggcatgccagcaaatgggaagaccatcgc) were used. Simultaneously, the GFP ORF was amplified from the Fire Lab vector *pPD95.75* using primers *CB90* (GCTTGCATGCCTGCAGGTCG) and *CB92* (AAGGGCCCGTACGGCCGACTAGTAGG). After gel purification of both PCR products, the two were mixed and used as template for a fusion PCR, using *CB164* (sequence above) and *CB91* (AAACAGTTATGTTTGGTATATTGGG). The primers for the fusion reaction were phosphorylated prior to the PCR, allowing the product to be ligated into the *Eco*RV site of *pAW400* to produce plasmid *pAW373*. This construct was modified to produce *pAW390*, in which GATA site A was deleted, and *pAW393*, in which GATA site B was deleted. *pAW390* was made by PCR, using phosphorylated primers *CB31* (aagaagtaggtggaaaagaaatgg) and *CB30* (ctctacgtaacgggaatgtgttg). *pAW393* was derived in a similar fashion, using primers *CB22* (tctagagtcggatctttgtccttgttag) and *CB60* (caatagatctaagggcgaattctg).

The mutagenesis of the *bro-1::dsRED2* construct, *pAW303* [10], to give *pAW305*, was performed by PCR, using phosphorylated primers *CB47* (atcgtatgtgtgttttcagactttc) and *CB46* (gcaaagagtgtatgttgttgtagg) flanking the 122bp CNE. After the PCR, the template was digested with *Dpn*I, and the PCR product purified and self-ligated.

To make plasmids *pAW545* (*bro-1* control intron::GFP) and *pAW546* (*bro-1* upstream sequence::GFP), the appropriate regions of *bro-1* were amplified with *CB150* (cccaagcttcaatttcgtcgaaaaaataacg) and *CB151* (ctctagagtttgagaatattcaccaagtgg), and *CB32* (aagcttgaaaccaaatcaaaatttagccc) and *CB33* (tctagagtagggctcaaatctgcagtaatac), respectively. Using the tags in the primers, these PCR products were digested with *HindIII* and *Xba*I. The minimal promoter plasmid *pPD107.94* was cut with the same enzymes and ligated with the inserts.

*pAW559* (*scm::rde-1cDNA*) was made in two stages. First, *rde-1* cDNA was amplified from a first strand cDNA preparation, using primers *CB289* (atgtcctcgaattttcccg) and *CB290* (ttatgcgaacgacattccag), and cloned into the *pCR®-XL-TOPO* vector (Invitrogen) creating *pAW570*, and sequenced. In parallel, the *scm* promoter was amplified from *pMF1* (*scm::gfp*) using primers *CB147* (aatgtctccttgtagtcctggtg) and *CB148* (gtccactctggaggcactatttc) and also cloned into *pCR®-XL-TOPO* vector, creating *pAW505*. Once the sequence of the cloned *rde-1* cDNA had been verified, it was re-amplified with phosphorylated primers and blunt-cloned into the *EcoRV* site of *pAW505*, giving *pAW559*.

*pAW549* (*eff-1* transcriptional reporter) was made in two stages. Firstly, a 4kb region of the *eff-1* promoter was amplified using primers *CB267* (ccagaacccaattgactgagg) and *CB266* (gcggttccatgcttctg) and cloned in the reverse direction into *pCR®-XL-TOPO* vector, creating *pAW571. pAW571* was then digested with *Bam*HI and *Pst*I; the promoter-containing fragment was ligated into *pPD95.75*, which had been cut with the same enzymes.

All constructs were checked by sequencing before use.

**Construction of Yeast strains**

3 copies of the 122bp *bro-1* CNE were inserted in the forward direction into the Clontech Matchmaker vectors *pHisi-1* and *pLacZi* by blunt-cloning the CNE PCR product (made using phosphorylated primers *CB1* (aagcttctatttcaacacattcccgttac) and *CB2* (agatctattgtaatcttgtcggatctttg)) into the vectors which were cut with *Sma*I. The plasmids were integrated into the yeast strain *YM4271* (*MATa*, *ura3-52*, *his3-∆200*, *ade2-101*, *lys2-801*, *leu2-3,* *trp1-901*, *tyr1-501*, *gal4-∆512*, *gal80-∆538*, *ade5::hisG* [57]) creating the double integrant strain *YM4271* [p*bro-1*HIS; p*bro-1*LAC], using the LiAc transformation procedure, as described in the Clontech Matchmaker manual.
